# Synthesis of jet fuel range branched cycloalkanes with mesityl oxide and 2-methylfuran from lignocellulose

**DOI:** 10.1038/srep32379

**Published:** 2016-09-01

**Authors:** Shanshan Li, Ning Li, Wentao Wang, Lin Li, Aiqin Wang, Xiaodong Wang, Tao Zhang

**Affiliations:** 1 State Key Laboratory of Catalysis, Dalian Institute of Chemical Physics, Chinese Academy of Sciences, Dalian 116023, China; 2 Graduate University of Chinese Academy of Sciences, Beijing 10049, China; 3 iChEM (Collaborative Innovation Centre of Chemistry for Energy Materials), Dalian Institute of Chemical Physics, Chinese Academy of Sciences, Dalian 116023, China

## Abstract

Jet fuel range branched cycloalkanes with high density (0.82 g mL^−1^) and low freezing point (217–219 K) was first prepared by the solvent-free intramolecular aldol condensation of the trione from the hydrolysis of the alkylation product of mesityl oxide and 2-methylfuran (or the one-pot reaction of mesityl oxide, 2-methylfuran and water), followed by hydrodeoxygenation (HDO).

In recent years, the synthesis of jet fuel range hydrocarbons with the lignocellulose derived platform compounds has drawn tremendous attention^1^,^2^,^3^,^4^,^5^,^6^,^7^,^8^,^9^,^10^,^11^,^12^,^13^. So far, most of the research work about lignocellulosic bio-jet fuel was concentrated on the synthesis of straight and branched chain alkanes. Compared with conventional jet fuel (a mixture of straight and branched chain alkanes, cycloalkanes and aromatics), these chain alkanes have lower density (~0.76 g mL^−1^) and volumetric heat values. As a solution to this problem, it is still imperative to develop new routes for synthesis of jet fuel range cycloalkanes and aromatics with lignocellulose derived platform chemicals^14^,^15^,^16^,^17^,^18^,^19^.

Mesityl oxide is the self aldol condensation product of acetone from the acetone-butanol-ethanol (ABE) fermentation of lignocellulose^20^,^21^. 2-Methylfuran (2-MF) is the selective hydrogenation product of furfural which has been manufactured on industrial scale by the hydrolysis-dehydration of the hemicellulose part of agriculture waste and forest residue^22^,^23^. In this work, a new route for the synthesis of jet fuel range branched cycloalkanes with high density and low freezing point was developed by the solvent-free intramolecular aldol condensation of the trione from the hydrolysis of the alkylation product of mesityl oxide and 2-MF, followed by hydrodeoxygenation (HDO) (see Fig. 1).

## Results and Discussion

### Hydrolysis reaction

**Figure 1:**
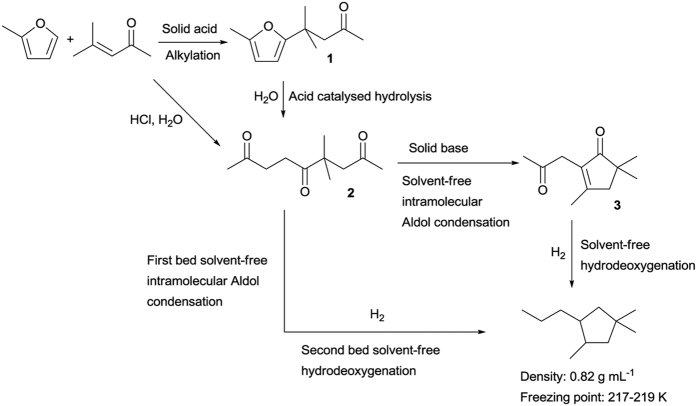
Reaction pathway for the synthesis of jet fuel range cycloalkanes with mesityl oxide and 2-methylfuran (2-MF).

**Figure 2:**
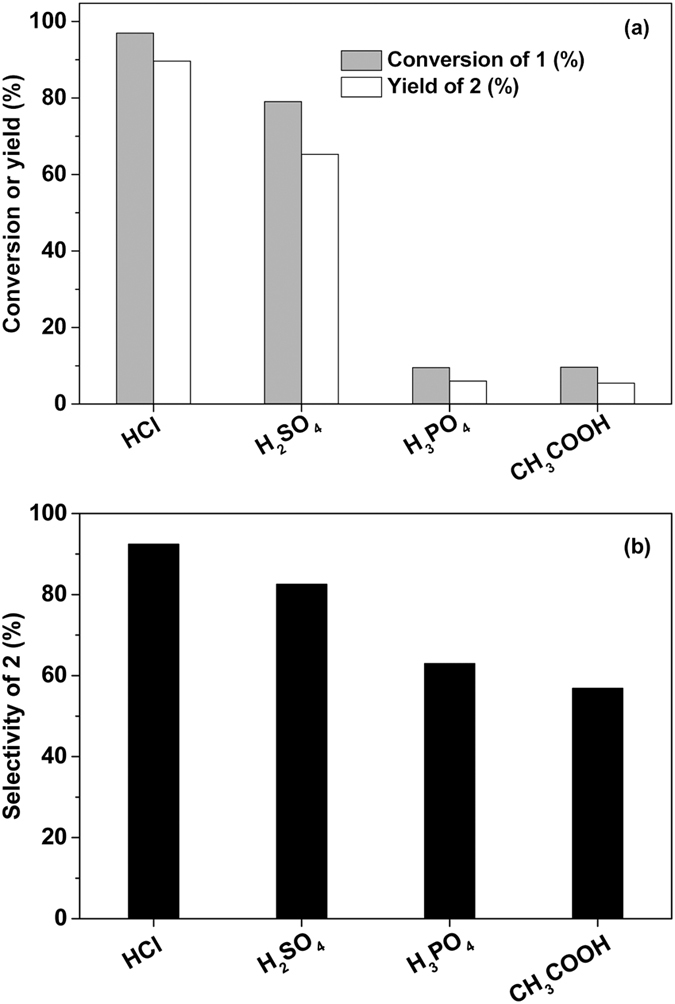
Hydrolysis of compound 1 to compound 2 under the catalysis of a series of liquid Brϕnsted acid catalysts. Reaction conditions: 1.0 g, 5.55 mmol compound **1**, 1 mL 6 N liquid acid solution; 333 K, 2 h.

**Figure 3:**
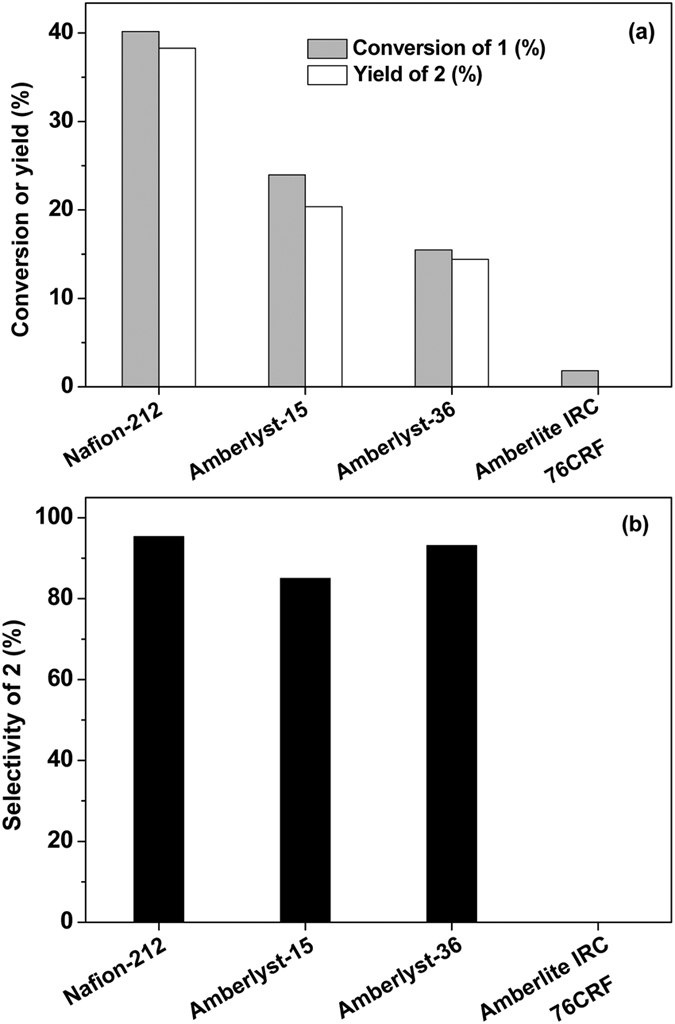
Hydrolysis of compound 1 to compound 2 under the catalysis of a series of acidic resins. Reaction conditions: 1.0 g, 5.55 mmol compound **1**, 1 mL H_2_O, 0.15 g solid acidic resin; 333 K, 2 h.

In the first step of this work, the 4-methyl-4-(5-methylfuran-2-yl)pentan-2-one (*i.e.* compound **1** in Fig. 1) obtained from the alkylation of mesityl oxide and 2-MF was hydrolysed to 4,4-dimethylnonane-2,5,8-trione (*i.e.* compound **2** in Fig. 1) under the promotion of acid catalysts. Among the Brϕnsted acids investigated in this work, HCl exhibited the highest activity and selectivity for the hydrolysis of compound **1** (see Fig. 2). Under the catalysis of HCl, high conversion of compound **1** (97%) and good carbon yield (~90%) or selectivity (92.4%) of compound **2** were obtained under mild conditions (333 K, 2 h). According to the p*Ka* values listed in Supplementary Table S1, the excellent performance of HCl can be explained by its higher acid strength. Some acidic resins (such as Nafion-212, Amberlyst-15, Amberlyst-36 and Amberlite IRC 76CRF) are also effective for the hydrolysis reaction, although their activities are relatively lower than that of HCl (see Fig. 3). The activity sequence of the acid resins is Nafion-212 > Amberlyst-15, Amberlyst-36 > Amberlite IRC 76CRF. This sequence is consistent with the acid strength sequence of these materials which is indicated by the initial NH_3_ adsorption heats illustrated in Supplementary Fig. S1 and Table S2. As we know, Nafion is a perfluorinated sulfonic acid resin^24^. Amberlyst is a sulfonic-acid-functionalized cross-linked poly-styrene resin^25^,^26^. Due to the electronic effect of fluorine, the acid strength of the SO_3_H groups on Nafion resin is higher than those on Amberlyst resin. In contrast, Amberlite IRC 76CRF is a weakly acidic cation exchange resin containing carboxyl group within a porous crosslink acrylic matrix^27^. Therefore, the acid strength of the Amberlite IRC 76CRF is lower than those of Nafion-212 and Amberlyst-15.

**Figure 4:**
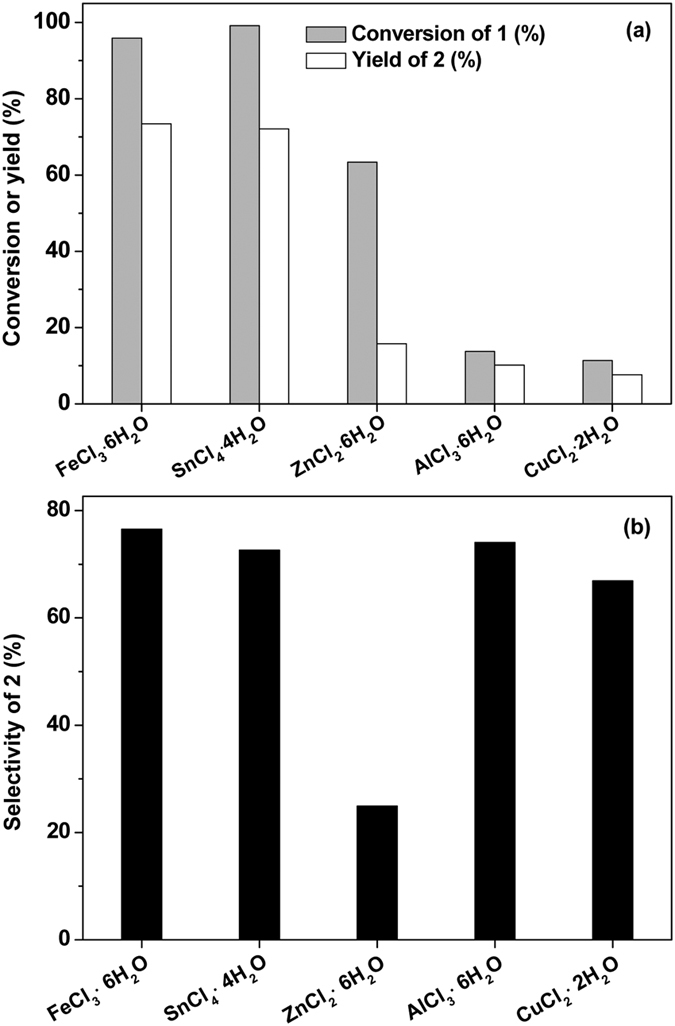
Hydrolysis of compound 1 to compound 2 under the catalysis of a series of Lewis acid catalysts. Reaction conditions: 1.0 g, 5.55 mmol compound **1**, 1 mL 6 mol L^−1^ Lewis acid solution; 333 K, 2 h.

The effect of acid type on the hydrolysis of compound **1** was investigated. From Fig. 4, we can see that Lewis acid catalysts (such as FeCl_3_, SnCl_4_, ZnCl_2_, AlCl_3_ and CuCl_2_) are also effective for the hydrolysis of compound **1**. Based on this result, we believe that the hydrolysis of compound **1** can be catalysed by both Brϕnsted acid and Lewis acid. Among the catalysts investigated in this work, HCl has the highest activity and good selectivity for the hydrolysis of compound **1**. After reaction, it can be easily separated from compound **2** and unreacted compound **1** (due to the lower solubility of compound **1** and **2** in water). Therefore, we think that HCl may be a promising catalyst for the hydrolysis of compound **1** in future application. To further explore the possibility for the industrial utilization of HCl, we also studied its recyclability in the hydrolysis of compound **1**. From Supplementary Fig. S2, we can see that the HCl solution is reusable under the investigated conditions. No evident decrease of activity or selectivity was observed after the HCl solution was repeatedly used for 3 times.

Taking into account that both the alkylation and the subsequent hydrolysis are acid catalysed reactions, we also explored the possibility to directly synthesize compound **2** with mesityl oxide and 2-MF. From the results shown in Supplementary Table S3, high carbon yield of compound **2** (up to 64.0%) can also be achieved under the catalysis of HCl solution by a two-stage reaction, which means that the alkylation and hydrolysis can be combined as a one-pot reaction. In real application, higher carbon yield of compound **2** can be obtained by further optimization of reaction conditions.

### Aldol condensation

By the solvent-free intramolecular aldol condensation of compound **2** under the promotion of some solid base catalysts, 3,5,5-trimethyl-2-(2-oxopropyl)cyclopent-2-enone (*i.e.* compound **3** in Fig. 1) was obtained as the main product. This compound can be used as a potential precursor for jet fuel range branched cycloalkane. To the best of our knowledge, this is the first report about the synthesis of precursor for jet fuel range cycloalkane without using the lignocellulose derived cyclic platform compounds (such as cyclopentanone^15^,^19^,^28^, cyclopentanol^16^, cyclohexanone^18^,^19^, phenols^14^ and aromatics^17^).

**Figure 5:**
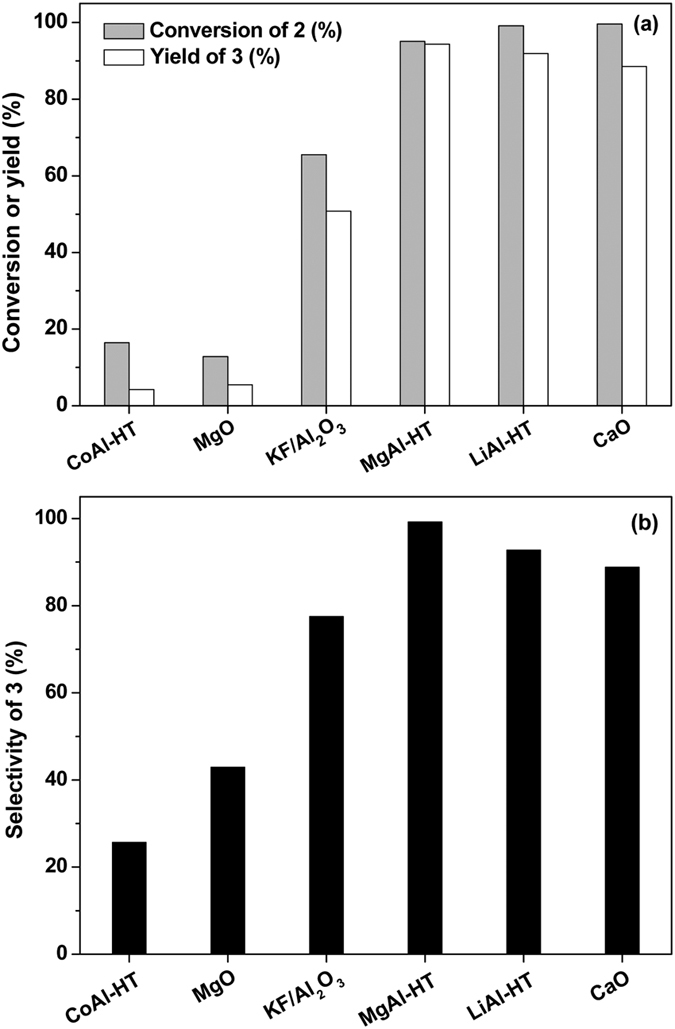
Solvent-free intramolecular aldol condensation of compound 2 to compound 3 over a series of solid base catalysts. Reaction conditions: 1.5 g, 7.57 mmol compound **2**, 0.2 g catalyst; 423 K, 6 h.

Among the investigated catalysts, MgAl-hydrotalcite (MgAl-HT) exhibited the best performance for the solvent-free intramolecular aldol condensation of compound **2** (see Fig. 5). Over it, high conversion of compound **2** (95.1%) and good carbon yield (94.4%) or selectivity (99.2%) of compound **3** can be achieved under mild conditions (423 K, 6 h). According to the literature about some similar reaction systems^13^,^15^,^29^,^30^ and the characterization results illustrated in Supplementary Table S4, Figs S3 and S4, the excellent performance of MgAl-HT catalyst can be rationalized by its bigger specific surface area, higher base strength or the higher amount of base sites and the weak acid sites on the surface of this catalyst.

### Hydrodeoxygenation (HDO)

**Figure 6:**
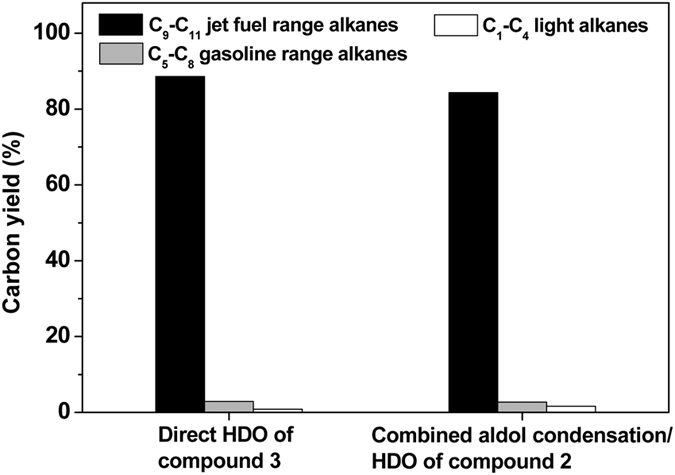
Carbon yields of C_9_-C_11_ jet fuel range alkanes, C_5_-C_8_ gasoline range alkanes and C_1_-C_4_ light alkanes from the HDO of compound 3 (or the combined aldol condensation/HDO of compound 2). Reaction conditions: 503 K, 0.5 MPa; 1.8 g Ni-SiO_2_ catalyst (or 1.0 g MgAl-HT catalyst for the first bed and 1.8 g Ni-SiO_2_ catalyst for the second bed); liquid feedstock flow rate 0.04 mL min^−1^; hydrogen flow rate: 120 mL min^−1^.

As the final aim of this work, the solvent-free HDO of compound **3** was carried out over the Ni-SiO_2_ catalyst prepared by the deposition-precipitation method developed by Lercher *et al*. in their previous work about the cleavage of ether bonds of lignin-derived aromatic ethers and hydrogenation of oxygen-containing intermediates at low temperatures^31^. From the results illustrated in Fig. 6, complete compound **3** conversion and high carbon yield (88.6%) of jet fuel range C_9_-C_11_ branched cycloalkanes can be obtained by the solvent-free HDO of compound **3** under mild conditions (503 K, 0.5 MPa H_2_). According to our measurement, such a mixture of C_9_-C_11_ branched cycloalkanes has a high density (0.82 g mL^−1^ at 293 K) and a low freezing point (219 K). As a potential application, they can be used as additives to improve the volumetric heat value of bio-jet fuel.

Furthermore, we also explored the possibility to directly synthesize jet fuel range cycloalkanes with compound **2** and hydrogen. The experiment was carried out in a dual-bed catalyst system (illustrated in Supplementary Fig. S5) under the same reaction conditions as we used for the solvent-free HDO of compound **3** (503 K, 0.5 MPa H_2_). In the first bed, the compound **2** was converted to compound **3** under the promotion of MgAl-HT catalyst. In the second bed, the compound **3** generated in the first bed was further hydrodeoxygenated to jet fuel range cyclic alkanes over the Ni-SiO_2_ catalyst. As we can see from Fig. 6, complete compound **2** conversion and high carbon yield (84.3%) of C_9_-C_11_ branched cycloalkanes can be obtained over the dual-bed catalyst system. The density (0.82 g mL^−1^ at 293 K) and freezing point (217 K) of the C_9_-C_11_ branched cycloalkanes as obtained were almost the same as the ones obtained by the direct HDO of compound **3** over the Ni-SiO_2_ catalyst (0.82 g mL^−1^ and 219 K). In real application, the dual-bed catalyst system is advantageous due to its higher efficiency and lower energy consumption.

## Conclusions

A new route was developed for the synthesis of jet-fuel range branched cycloalkanes with mesityl oxide and 2-MF which can be obtained from lignocellulose. The jet fuel range branched cycloalkanes obtained in this work have high density and low freezing point, simultaneously. As a potential application, they can be used as additive to improve the volumetric heat value of current bio-jet fuel. This work offers a potential solution to the shortage of current bio-jet fuel at density and volumetric heat value.

## Methods

### Preparation of catalysts

The Nafion-212, Amberlyst-15, Amberlyst-36 resins, CaO and MgO used in this work are commercial available. The KF/Al_2_O_3_ catalyst was obtained by the incipient wetness impregnation of γ-Al_2_O_3_ with an aqueous solution of KF, followed by drying at 393 K for 6 h and calcination at 873 K for 4 h in N_2_ flow. The KF content in the KF/Al_2_O_3_ catalyst is 40 wt.%. The MgAl-hydrotalcite (MgAl-HT), LiAl-hydrotalcite (LiAl-HT) and CoAl-hydrotalcite (CoAl-HT) were prepared by the co-precipitation methods described in our previous work^30^. The characterization results of the solid acid and solid base catalysts were given in the supporting information. The Ni-SiO_2_ catalyst used in the HDO process was obtained by the deposition-precipitation method described by Lercher *et al*.^31^. The theoretical Ni content in the Ni-SiO_2_ was controlled as 40 wt.%.

### Activity test

The acid-catalysed hydrolysis of compound **1** was conducted in a flask at 333 K for 2 h. The solvent-free intramolecular aldol condensation of compound **2** was carried out in a Teflon lined batch reactor. After reaction, the products were taken out from the reactor, filtrated and analysed by an Agilent 7890A GC. The detail information for the activity tests, the preparation and purification of compound **1**, **2** and **3** was described in supplementary information.

The solvent-free HDO of compound **3** was carried out in a stainless steel tubular flow reactor described in our previous work^32^. Before the HDO process, the Ni-SiO_2_ catalyst was reduced *in-situ* by hydrogen flow at 773 K for 2 h. After the reactor was cooled down to 503 K and stabilized for 0.5 h, the purified compound **3** was pumped into the reactor at 0.04 mL min^−1^ from the top of the reactor along with hydrogen at a flow rate of 120 mL min^−1^. After coming out from the tubular reactor, the products became two phases in a gas-liquid separator. The gaseous products flowed through a back pressure regulator to maintain the system pressure at 0.5 MPa and were analysed by an Agilent 6890N GC. The liquid products were drained periodically from the gas-liquid separator and analysed by an Agilent 7890A GC. The combined aldol condensation/HDO process of compound **2** was carried out in the same reactor as we used for the HDO of compound **3** over a dual-bed catalyst system (see Supplementary Fig. S5). In the first bed, the compound **2** was converted to compound **3** by the solvent-free aldol condensation over MgAl-HT catalyst. In the second bed, the compound **3** generated in the first bed was further hydrodeoxygenated to cyclic alkanes over the Ni-SiO_2_ catalyst. To facilitate the comparison, the reaction conditions (including the reaction temperature, hydrogen pressure, the rates of hydrogen and liquid feedstocks) are exactly the same as the ones we used for the HDO of compound **3**.

## Additional Information

**How to cite this article**: Li, S. *et al*. Synthesis of jet fuel range branched cycloalkanes with mesityl oxide and 2-methylfuran from lignocellulose. *Sci. Rep.* **6**, 32379; doi: 10.1038/srep32379 (2016).

## Supplementary Material

Supplementary Information
