# Supplementary material for: Synthesis of jet fuel range branched cycloalkanes with mesityl oxide and 2-methylfuran from lignocellulose
Source: Sci Rep. 2016 Sep 1;6:32379. doi: 10.1038/srep32379 (PMC5007666; doi:10.1038/srep32379)
Supplement: Supplementary Information [file srep32379-s1.pdf]

Supporting information

Synthesis of jet fuel range branched cycloalkanes with mesityl oxide and 2-methylfuran from lignocellulose

Shanshan Li,[1,2] Ning Li,*[1,3] Wentao Wang,[1] Lin Li,[1] Aiqin Wang,[1,3] Xiaodong Wang,[1] and Tao Zhang*[1,3]

[1] *State Key Laboratory of Catalysis, Dalian Institute of Chemical Physics, Chinese Academy of Sciences, Dalian 116023, China.*

[2] *Graduate University of Chinese Academy of Sciences, Beijing 10049, China.*

[3] *iChEM (Collaborative Innovation Centre of Chemistry for Energy Materials), Dalian Institute of Chemical Physics, Chinese Academy of Sciences, Dalian 116023, China.*

*Corresponding author E-mail: taozhang@dicp.ac.cn (T. Zhang) or lining@dicp.ac.cn (N. Li)

Preparation of 4-methyl-4-(5-methylfuran-2-yl)pentan-2-one:

4-Methyl-4-(5-methylfuran-2-yl)pentan-2-one (*i.e.* compound **1** in Fig. 1) was prepared by the alkylation of 2-methylfuran and mesityl oxide under the catalysis of Nafion-212 resin. The reaction was carried out in a round bottom flask which was put in a water bath. Typically, 16.4 g, 200 mmol 2-methylfuran (2-MF), 19.6 g, 200 mmol mesityl oxdie and 0.5 g catalyst were put into the round-bottom flask. The mixture was stirred at 303 K for 13 h. After being cooled down to room temperature, the liquid product was filtered and purified by the vacuum distillation. According to our analysis, high 2-MF conversion (61.4%) and carbon yield of compound **1** (56.8%) were achieved under the investigated conditions. The selectivity of compound **1** was calculated as 92.5%. The compound **1** as obtained (existing as a colorless liquid at room temperature) was stored in a refrigerator before being used in the subsequent hydrolysis tests.

According to the ^{13}C and ^{1}H NMR spectra shown in Supplementary Fig. S6, the purified compound **1** has high purity. Its chemical composition (70.83wt.% C, 8.53wt.% H, 20.64wt.% O) measured by an Elementar Vario EL III (Elementar) is very close to the theoretical one (73.3wt.% C, 8.95wt.% H, 17.75wt.% O).

Activity tests

Hydrolysis

The hydrolysis of compound **1** to 4,4-dimethylnonane-2,5,8-trione (*i.e.* compound **2** in Fig. 1) was conducted in a flask at 333 K for 2 h with acid solutions or solid acids as catalysts. For liquid Brϕnsted acid catalysts, 1 mL acid

solution and 1.0 g, 5.55 mmol compound **1** were used for each test. To facilitate the comparison, the concentrations of HCl, H_2SO_4, H_3PO_4 and CH_3COOH were controlled as 6 mol L^{-1}, 3 mol L^{-1}, 2 mol L^{-1} and 6 mol L^{-1} (*i.e.* 6 N), respectively. For solid acid catalysts (such as Nafion-212, Amberlyst-15, Amberlyst-36 and Amberlite IRC 76CRF resins), 0.15 g catalyst, 1.0 g, 5.55 mmol compound **1** and 1.0 g, 55.51 mmol water were used for each test. For Lewis acid catalysts, 1 mL Lewis acid solution and 1.0 g, 5.55 mmol compound **1** were used for each test. To facilitate the comparison, the concentrations of the Lewis acid solutions were controlled as 6 mol L^{-1}. After the reaction, the products was analysed by an Agilent 7890 gas chromatograph (GC) equipped with a HP-5 capillary column (30 m, 0.25 mm I.D., 0.5 μm film) and Flame Ionization Detector (FID).

The compound **2** (existing as a yellow liquid at room temperature) used in the solvent free intramolecular aldol condensation was prepared by the hydrolysis of compound **1** catalysed by HCl solution. Typically, 25 g, 138.8 mmol compound **1** and 25 mL HCl solution (6 mol L^{-1}) were used. The mixture was stirred at 333 K for 2 h. After being cooled down to room temperature, the organic phase was separated from HCl solution by a separating funnel, pretreated with Na_2CO_3 solution (to remove the HCl solved in hydrolysis product of compound **1**) and extracted with methylene chloride. Finally, the compound **2** was obtained after removal of solvent by vacuum distillation at 303 K. According to the ^{13}C and ^{1}H NMR spectra illustrated in Supplementary Fig. S7, the compound **2** as obtained also has high purity. Its chemical composition (65.50wt.% C, 9.02wt.% H,

25.48wt.% O) measured by an Elementar Vario EL III (Elementar) is very close to the theoretical one (66.64wt.% C, 9.15wt.% H, 24.21wt.% O).

One-pot alkylation/hydrolysis

To fulfil the need of real application, we also explored the possibility to directly synthesize compound **2** with mesityl oxide and 2-MF by combining the alkylation step and the subsequent hydrolysis step into one-pot reaction. Typically, 1.64 g, 20 mmol 2-MF, 1.92 g, 20 mmol mesityl oxide and 1 mL HCl (37wt.%) were used in the tests. The mixture was stirred at different temperatures for 4 h. After the reaction, the products was analysed by an Agilent 7890 GC equipped with a HP-5 capillary column (30 m, 0.25 mm I.D., 0.5 μm film) and FID detector. To further increase the carbon yield and selectivity of target product (*i.e.* compound **2)**, we also explored the direct synthesis of compound **2** by a two-stage process. To do this, the reaction was carried out at 273 K for 4 h, then at 301 K for 2 (or 4) h.

Solvent-free aldol condensation

The solvent-free intramolecular aldol condensation of compound **2** to 3,5,5-trimethyl-2-(2-oxopropyl)cyclopent-2-enone (*i.e.* compound **3** in Fig. 1) was carried out in a batch reactor. Typically, 1.5 g, 7.57 mmol purified compound **2** and 0.2 g catalyst were used for each test. The reaction was carried out at 423 K for 6 h. After that, the products were taken out from the reactor, filtrated and analysed by an Agilent 7890 GC equipped with a HP-5 capillary column (30 m, 0.25 mm I.D., 0.5 μm film) and FID detector.

The compound **3** used in the solvent-free hydrodeoxygenation (HDO) step was

prepared by the solvent-free intramolecular aldol condensation of 30 g, 151.4 mmol purified compound **2** under the catalysis of 4.0 g MgAl-HT catalyst. The reaction was carried out at 423 K for 6 h. After the reaction, the compound **3** as obtained (existing as a dark orange liquid at room temperature) was further purified by the removal of MgAl-HT catalyst by filtration, extraction with methylene chloride and removal of solvent by vacuum distillation at 303 K. According to the high resolution mass spectrum (HRMS), ^{13}C and ^{1}H NMR spectra shown in Supplementary Fig. S8 and Fig. S9, the compound **3** as obtained has high purity. Its chemical composition (70.79wt.% C, 8.47wt.% H, 20.74wt.% O) measured by an Elementar Vario EL III (Elementar) is very close to the theoretical one (73.3wt.% C, 8.95wt.% H, 17.75wt.% O).

Carbon yields of different alkanes:

The carbon yields of different alkanes in the direct HDO of compound **3** or the combined aldol condensation/HDO process of compound **2** were calculated according to the following formulas:

Carbon yield of jet fuel range C_9-C_{11} alkanes (%) = Total carbon of C_9-C_{11} alkanes in the liquid products/Total carbon of feedstock pumped into the reactor × 100%

Carbon yield of gasoline range C_5-C_8 alkanes (%) = Total carbon of C_5-C_8 alkanes in the gas products per unit time/Total carbon of feedstock pumped into the reactor per unit time × 100% + Total carbon of the C_5-C_8 alkanes in liquid phase products/Total carbon of feedstock pumped into the reactor × 100%

Carbon yield of C_1-C_4 light alkanes (%) = Total carbon of the C_1-C_4 alkanes in the gas

products per unit time/Total carbon of feedstock pumped into the reactor per unit time $\times$100%

The density and freezing point of the jet fuel range C_9-C_{11} alkanes as obtained were measured by Anton Paar DMA4500M densitometer and PHASE PSA-70Xi Automatic freezing point instrument, respectively.

Characterization of catalysts

N_2-physisorption

The specific BET surface areas (S_{BET}) of the catalysts were measured by nitrogen physisorption at 77 K using an ASAP 2010 apparatus. Before each measurement, the sample was evacuated at 573 K for 3 h (for acid resins, the desorption temperature was set as 393 K). From the results listed in the Supplementary Table S2 and Table S4, there is no clear relationship between the specific surface areas of solid acids (or solid bases) investigated in this work and their performances in the hydrolysis of compound **1** (or the solvent-free intramolecular aldol condensation of compound **2**).

Chemical titration

The amounts of acid sites on the surfaces of solid acid catalysts were measured by chemical titration. Typically, 0.1 g catalyst was dispersed into 20 mL 2 mol L^{-1} NaCl solution, sonicated for 1.5 h, and then centrifuged. The resulting liquid was titrated with 8 mmol L^{-1} NaOH solution (pre-calibrated with a standard potassium hydrogenphthalate solution) using phenolphthalein as indicator. The molar amount of acid sites per gram of catalyst was calculated according to consumption of NaOH solution. From the results listed in the Supplementary Table S2, there is no clear

relationship between the amounts of acid sites on the surface of solid acids and their performances in the hydrolysis of compound **1**.

Microcalorimetric measurement of NH_3 adsorption

The microcalorimetric measurements of ammonia adsorption for the different solid acid catalysts used in the hydrolysis reaction were performed at 353 K using a BT2.15 heat-flux calorimeter (France, Seteram) connected to a gas-handling and a volumetric system employing MKS Baratron Capacitance Manometers for precision pressure measurement ($\pm 0.5 \times 10^{-4}$ Torr). Before each measurement, ammonia (purity > 99.9%) was purified by successive freeze-pump-thaw cycles. The specific mass of sample with similar amount of acid sites was evacuated in a quartz cell at 353 K overnight under high vacuum to remove the physically adsorbed substance. The differential heat was measured as a function of acid site coverage by repeatedly introducing small dosage of ammonia onto the samples until the equilibrium pressure reached about 5-6 torr. Then the system was evacuated overnight to remove the physically adsorbed ammonia, and a second adsorption was performed. The amount of irreversible adsorbed ammonia was determined by the difference between the isotherms of the first and second adsorption cycles. From the results illustrated in Supplementary Table S2 and , the initial NH_3 adsorption heats over different solid acids decrease in the order of Nafion-212 (168 kJ mol^{-1}) > Amberlyst-15 (156 kJ mol^{-1}), Amberlyst-36 (155 kJ mol^{-1}) > Amberlite IRC 76CRF (1.54 kJ mol^{-1}), which is consistent with their activity sequence in the hydrolysis of compound **1**.

CO_2 chemisorption and CO_2-temperature-programmed desorption (TPD)

The basicity of the solid base catalysts used in aldol condensation was characterized by CO_2 chemisorption and CO_2 temperature-programmed desorption (CO_2-TPD) experiments which were carried out on a Micromeritics AutoChem II 2920 Automated Catalyst Characterization System. For each test, 0.1 g sample was used. The catalyst was placed in a quartz reactor, pretreated in He flow at its preparation temperature for 1 h (for MgO and CaO, the samples were pretreated at 973 K and 723 K, respectively) and cooled down in He flow to 353 K. After the stabilization of base line, pulses of CO_2 (1 mL) were dosed in the reactor until saturation. The amounts of base sites on different catalysts were calculated by the adsorptions of CO_2 during the tests. After the saturated adsorption of CO_2, the sample was purged with He at 353 K for 45 min to remove the physically adsorbed CO_2. The desorption of CO_2 was carried out in He flow from 353 K to 1173 K at a heating rate of 10 K min^{-1}. The desorbed CO_2 molecules were detected by an OminiStar mass spectrometer (MS) equipped with the software quadstar 32-bit.

The CO_2 chemisorption results were shown in Supplementary Table S4. From it, we can see that the amounts of base sites over the solid base catalysts decrease in the order of MgAl-HT > LiAl-HT > CaO ~ CoAl-HT > KF/Al_2O_3, MgO.

The CO_2-TPD results were illustrated in Supplementary Fig. S3. In the CO_2-TPD profiles of the MgO and CoAl-HT catalysts, only broad CO_2 desorption peaks centred at low temperature were observed. Compared with them, the base strength of the KF/Al_2O_3 catalyst is higher because a CO_2 desorption peak was observed at 780 K. In

the CO_2-TPD profiles of the MgAl-HT, LiAl-HT and CaO catalysts, evident CO_2 desorption peaks at higher temperatures (> 850 K) were observed, indicating that these materials have higher base strength than other catalysts. According to the desorption temperatures and the amounts of CO_2 desorbed from the solid base catalysts, the base strength of different solid base catalysts decreases in the order of MgAl-HT ~ LiAl-HT > CaO > KF/Al_2O_3 > MgO, CoAl-HT.

NH_3 chemisorption and NH_3-temperature-programmed desorption (TPD)

The acidity of the solid base catalysts was measured by NH_3 chemisorption and NH_3 temperature-programmed desorption (NH_3-TPD) which were carried out on a Micrometeritics AutoChem II 2920 Automated Catalyst Characterization System. For each test, 0.1 g sample was used. Before the measurement, the sample was pretreated at 393 K for 2 h and cooled down to 373 K in He flow. After the stabilization of the base line, pulses of NH_3 (1 mL) were dosed in the reactor until saturation. The amounts of acid sites on different catalysts were calculated by the adsorptions of NH_3 during the tests. After the saturated adsorption of NH_3 at 373 K, the sample was kept at 373 K in He flow for 45 min to remove the physically adsorbed ammonia. The desorption of NH_3 was conducted in He flow from 373 K to 1073 K at a heating rate of 10 K min^{-1}. The desorbed NH_3 molecules were detected by an OminiStar mass spectrometry (MS) equipped with the software quadstar 32-bit.

According to the NH_3 chemisorption results shown in Supplementary Table S4, the amount of acid sites on the investigated solid base catalysts decrease in the order of CoAl-HT > MgAl-HT > LiAl-HT > KF/Al_2O_3 > CaO, MgO.

From Supplementary Fig. S4, we can see that NH_3-TPD profiles of the MgAl-HT, LiAl-HT and CoAl-HT catalysts only have low NH_3 desorption peaks centred at 450 K. Compared with the hydrotalcite catalysts, KF/Al_2O_3 has higher acid strength because a NH_3 desorption peak appeared at 850 K. However, the intensity of this peak is much lower than those of hydrotalcite catalysts. Compared with KF/Al_2O_3, the NH_3 desorption peaks were even lower in the NH_3-TPD profiles of MgO and CaO.

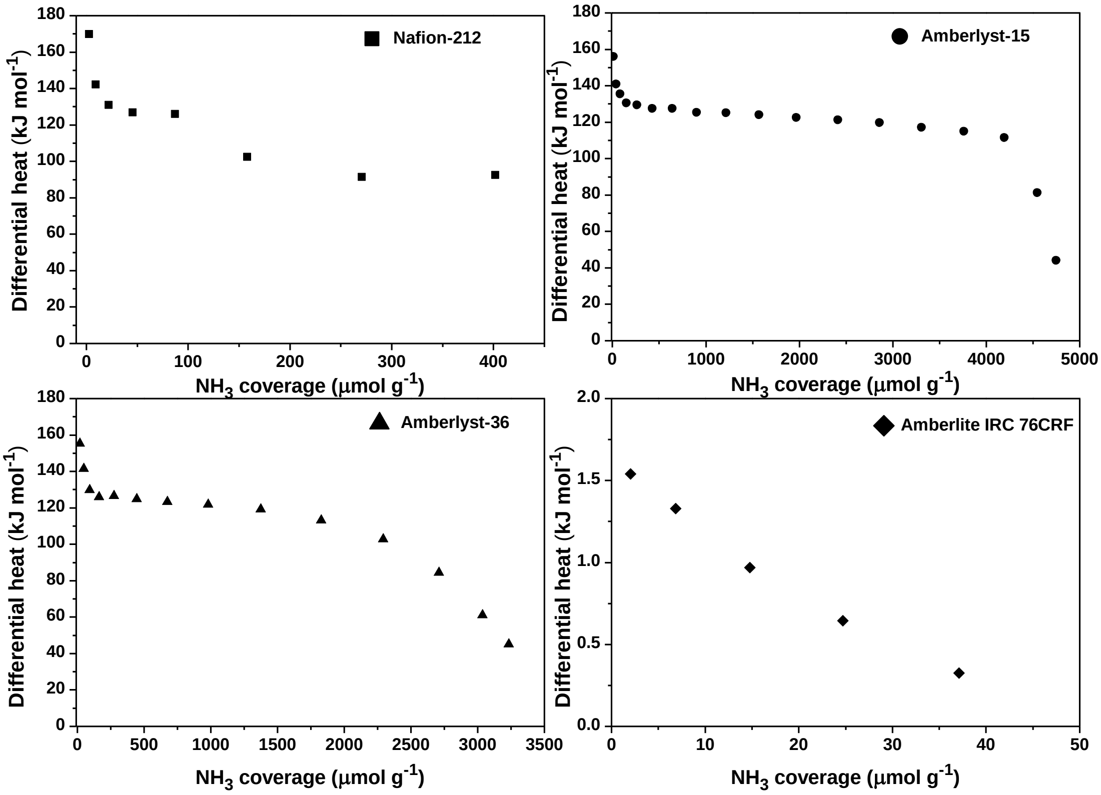

Figure S1 | Adsorption heat versus NH_3 coverage at 353 K on Nafion-212 (■), Amberlyst-15 (●), Amberlyst-36 (▲) and Amberlite IRC 76CRF (◆) resins.

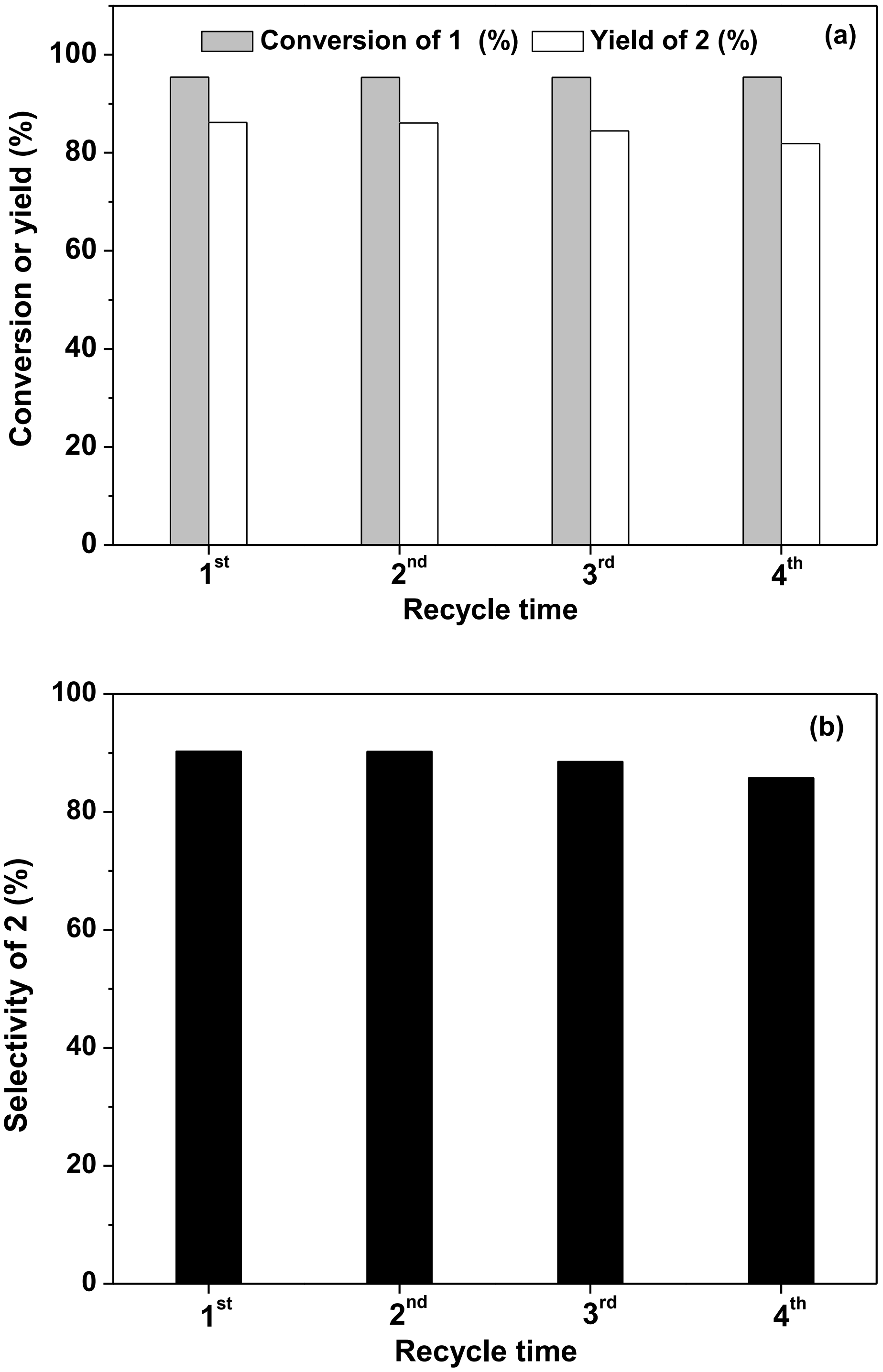

Figure S2 | Hydrolysis of compound 1 to compound 2 under the catalysis of HCl solution. Reaction conditions: 1.0 g, 5.55 mmol compound **1**, 1 mL 6 mol L^{-1} HCl solution; 333 K, 2 h.

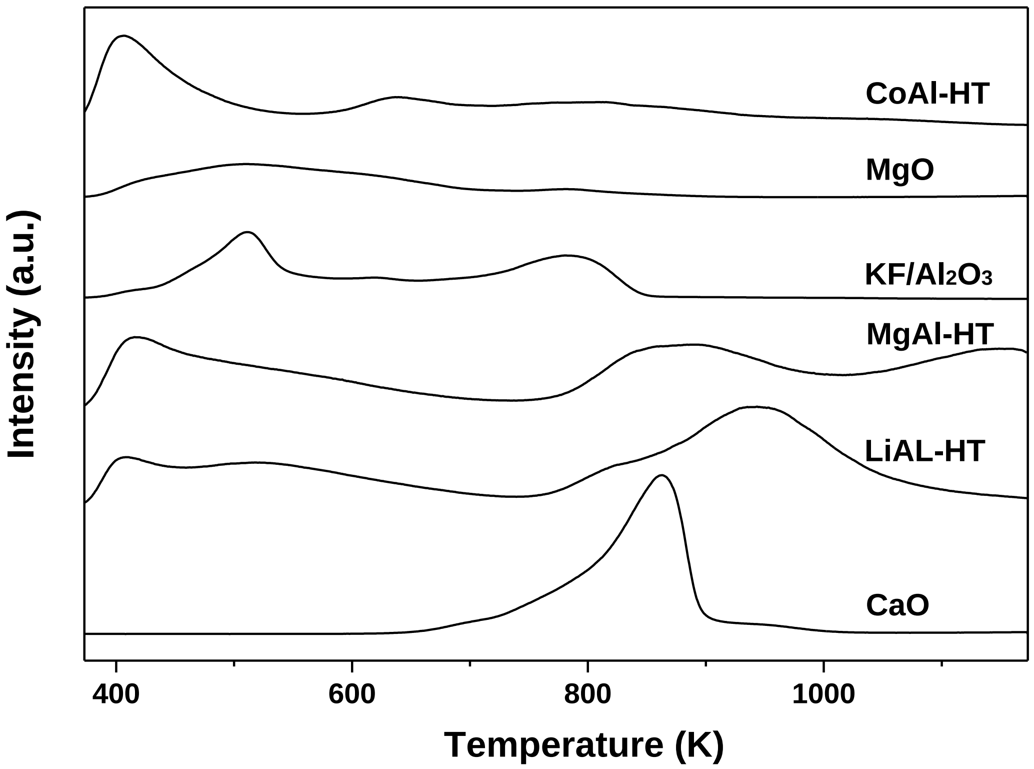

Figure S3 | CO_2-TPD profiles of the investigated solid base catalysts.

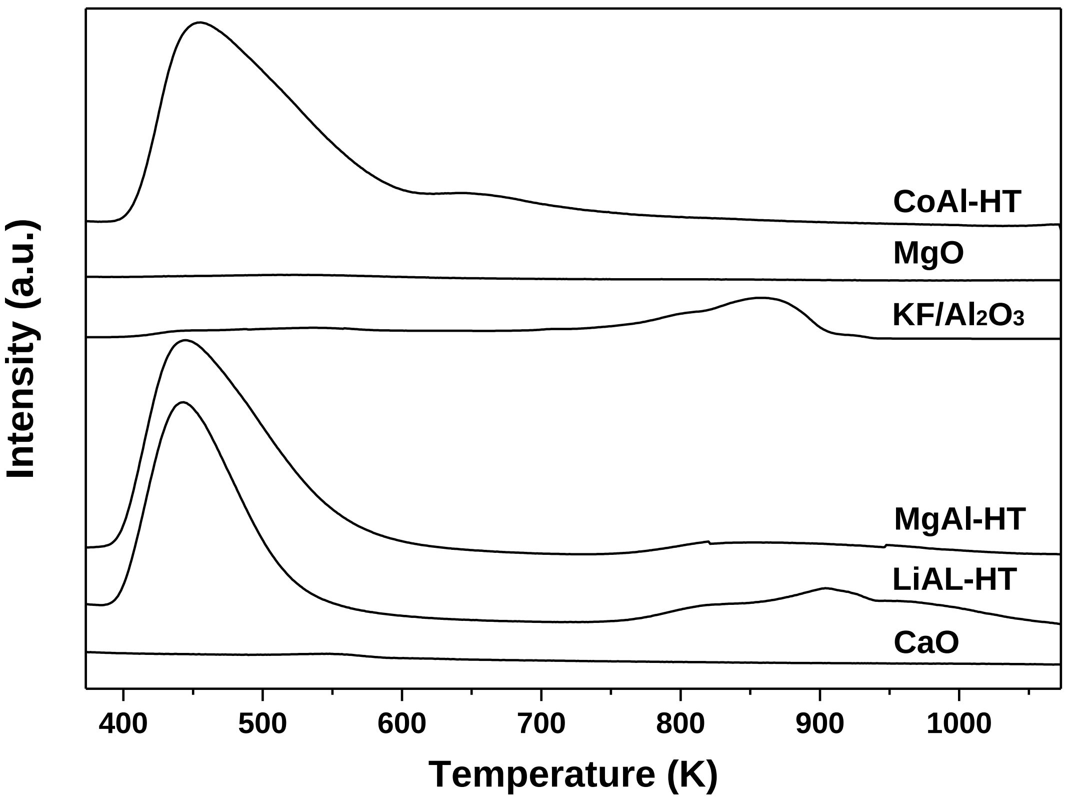

Figure S4 | NH_3-TPD profiles of the investigated solid base catalysts.

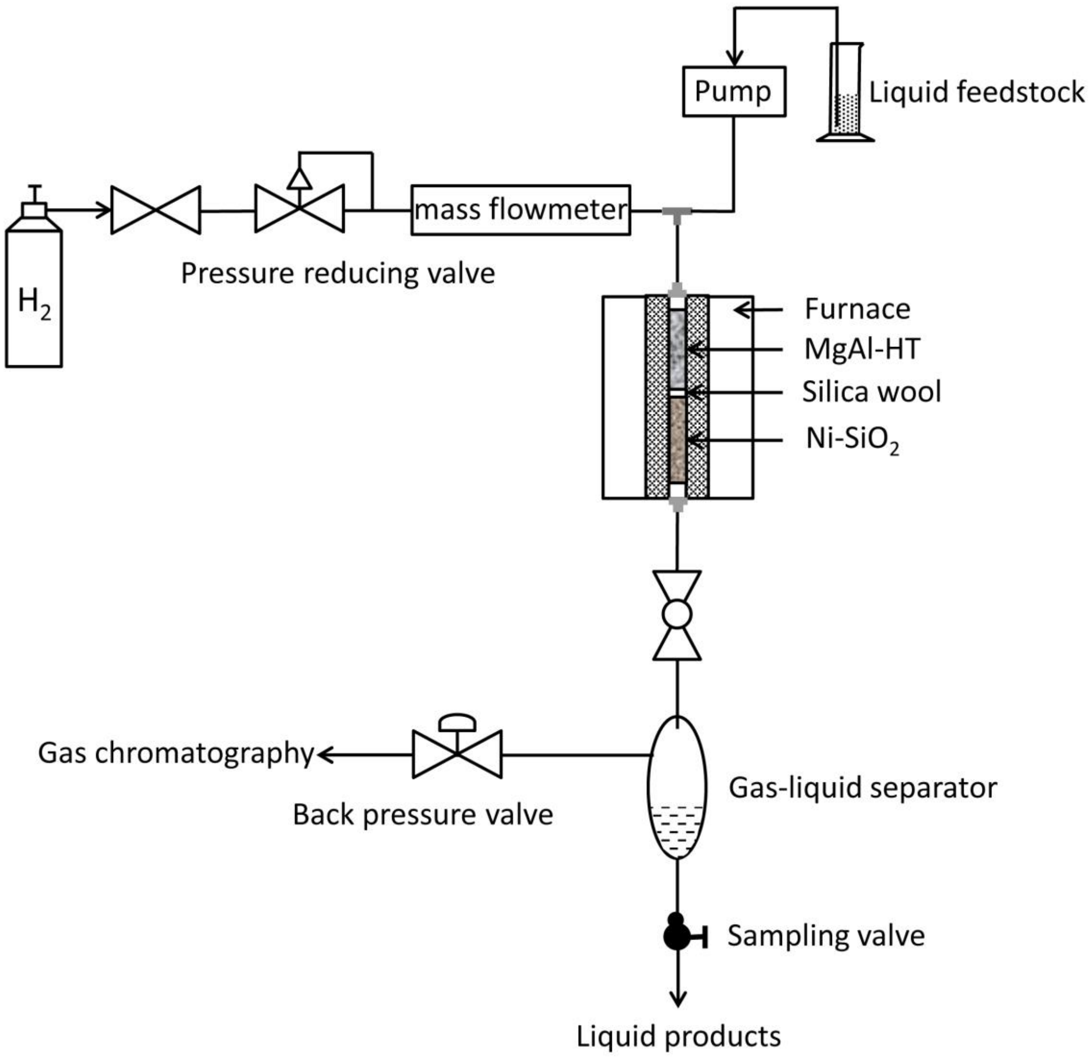

Figure S5 | Diagram of the dual-bed catalyst system used in the direct synthesis of jet fuel range cycloalkanes by the combined aldol condensation/HDO of compound 2.

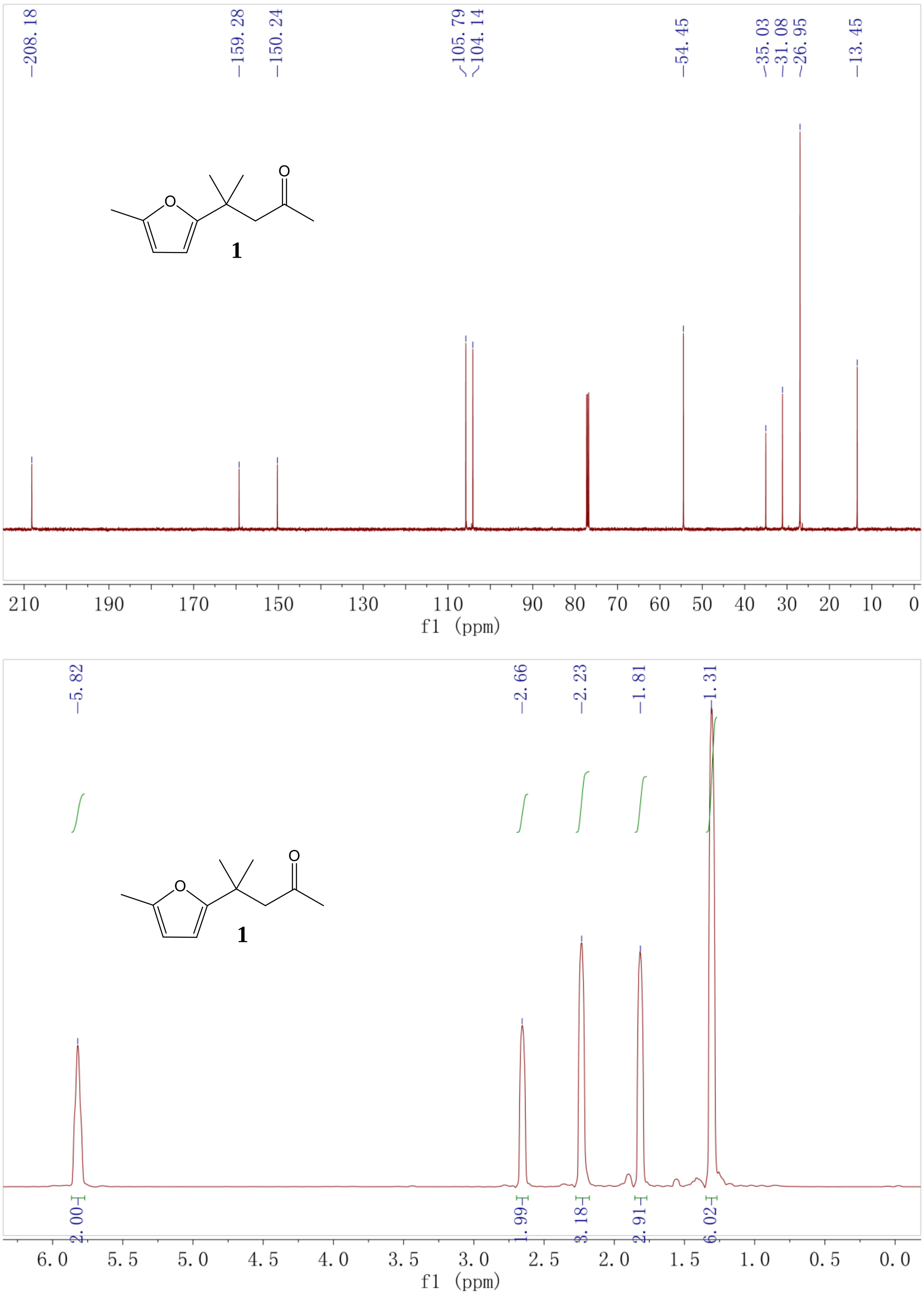

Figure S6 | ^{13}C and ^{1}H NMR spectra of the compound 1 from the alkylation of 2-methylfuran and mesityl oxide.

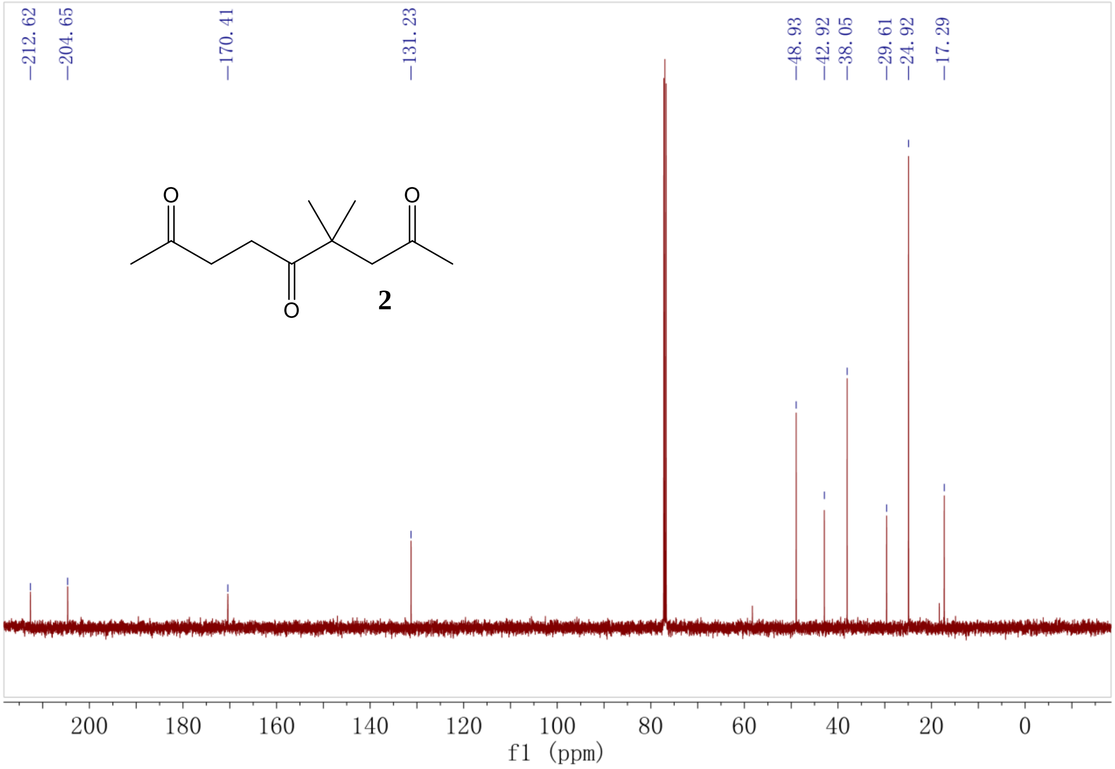

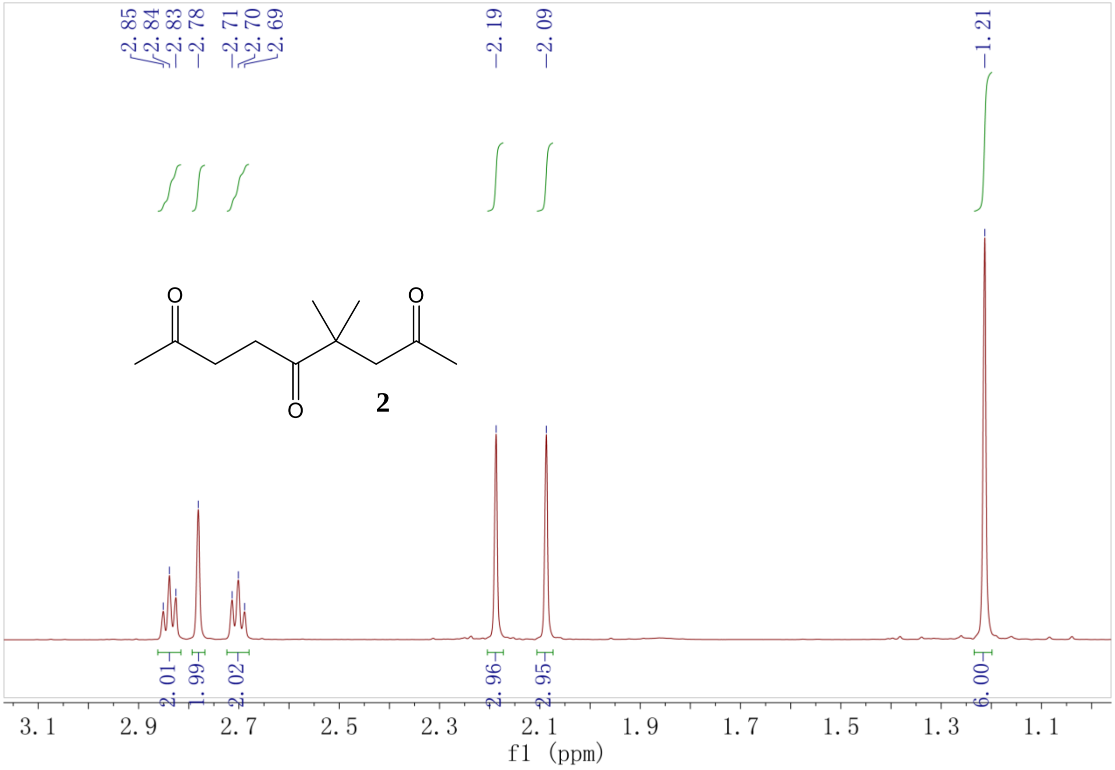

Figure S7 | ^{13}C and ^{1}H NMR spectra of the compound 2 from the hydrolysis of compound 1.

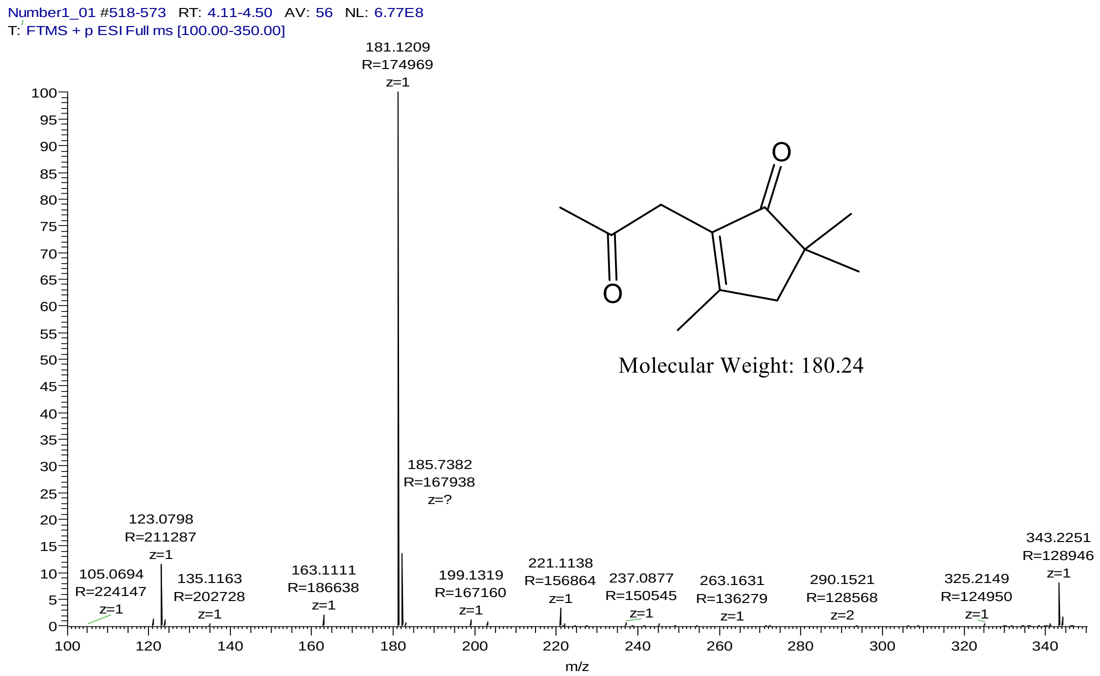

ESI-HRMS: calcd for $C_{11}H_{16}NO_2^+$ [M+H$^+$] 181.1223, found 181.1209.

Figure S8 | High resolution mass spectrum (HRMS) of compound 3 from the intramolecular self aldol condensation of compound 2.

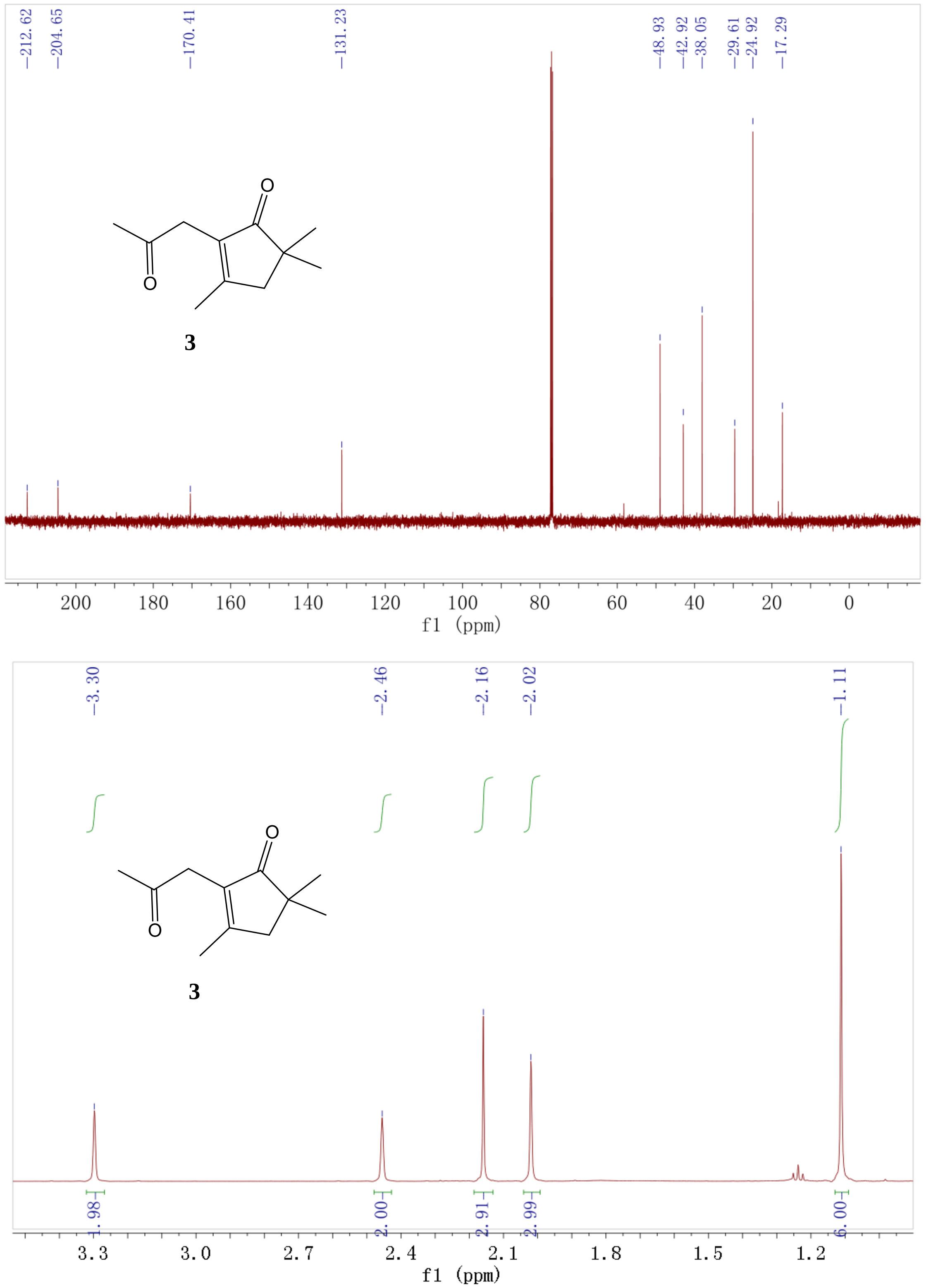

Figure S9 | ^{13}C and ^{1}H NMR spectra of compound 3 from the intramolecular self aldol condensation of compound 2.

Table S1 | The p*Ka* values of the Brϕnsted acids which were used in the hydrolysis of compound **1**.

Catalyst	p*Ka*
HCl	1.0[1]
H_2SO_4	1.99[2]
H_3PO_4	2.16[2]
CH_3COOH	4.76[2]

Table S2 | Specific BET surface areas (S_{BET}) and the amount of acid sites on the surfaces of the investigated solid acid catalysts.

Catalyst	S_{BET} (m^2 g^{-1}) [a]	Acid sites (mmol g^{-1}) [b]	Initial NH_3 adsorption heat (kJ mol^{-1}) [c]
Nafion-212	2	1.08	168
Amberlyst-15	41	4.61	156
Amberlyst-36	13	5.33	155
Amberlite IRC 76CRF	0.07	7.02	1.54

[a] Measured by N_2-physisorption.

[b] Measured by chemical titration.

[c] Measured by the microcalorimetric measurements of ammonia adsorption.

Table S3 | Conversion of 2-methylfuran (2-MF), carbon yields of compound **1** and compound **2** under the catalysis of 37wt.% HCl solution. Reaction conditions: 1.64 g, 20 mmol 2-MF, 1.92 g, 20 mmol mesityl oxide and 1 mL HCl (37wt.%). Before analysis, the HCl was neutralized with $NaHCO_3$ in ice-water bath.

Entry	Temperature (K)	Time (h)	Conversion of 2-MF (%)	Yield of compound **1** (%)	Yield of compound **2** (%)	Selectivity of compound **2** (%)
1	273	4	85.4	38.0	22.1	25.9
2	301	4	99.6	13.7	47.5	47.7
3	313	4	99.7	2.1	38.9	39.0
4 [a]	273<br>301	4<br>2	100	1.6	56.2	56.2
5 [b]	273<br>301	4<br>4	100	1.7	52.7	52.7
6 [c]	273<br>301	4<br>2	100	1.8	64.0	64.0

a: The reaction was carried out at 273 K for 4 h, then at 301 K for another 2 h.

b: The reaction was carried out at 273 K for 4 h, then at 301 K for another 4 h.

c: The reaction conditions were same as entry 5, but 1.7 mL HCl solution was used as the catalyst.

Table S4 | Specific BET surface areas (S_{BET}) and the amounts of base (or acid) sites on the surfaces of different solid base catalysts

Catalyst	S_{BET} (m^2 g^{-1}) [a]	Base sites (mmol g^{-1}) [b]	Acid sites (mmol g^{-1}) [c]
CoAl-HT	238	0.13	0.45
MgO	56	0.08	0.02
KF/Al_2O_3	215	0.09	0.05
MgAl-HT	241	0.21	0.38
LiAl-HT	242	0.18	0.26
CaO	18	0.14	0.03

[a] Measured by N_2-physisorption.

[b] Measured by CO_2-chemisorption.

[c] Measured by NH_3-chemisorption.

References

1. Eifert, J. D., Hackney, C. R., Pierson, M. D., Duncan, S. E. & Eigel, W. N. Acetic, Lactic, and Hydrochloric Acid Effects on Staphylococcus aureus 196E Growth Based on a Predictive Model. *Journal of Food Science* **62**, 174-178 (1997).
2. Lide, D. R. (ed). *CRC Handbook of Chemistry and Physics, 89th Edition (Internet Version 2009)*. CRC Press/Taylor and Francis: Boca Raton, FL, 2009.
